# The Mechanism by Which Dodecyl Dimethyl Benzyl Ammonium Chloride Increased the Toxicity of Chlorpyrifos to *Spodoptera exigua*

**DOI:** 10.3389/fphar.2017.00475

**Published:** 2017-07-18

**Authors:** Li Cui, Huizhu Yuan, Daibin Yang, Changhui Rui, Wei Mu

**Affiliations:** ^1^Key Laboratory of Integrated Pest Management in Crops, Ministry of Agriculture, Institute of Plant Protection, Chinese Academy of Agricultural Sciences Beijing, China; ^2^Key Laboratory of Pesticide Toxicology and Application Technique, Shandong Provincial Key Laboratory for Biology of Vegetable Diseases and Insect Pests, College of Plant Protection, Shandong Agricultural University Tai'an, China

**Keywords:** mechanism, cationic surfactant DDBAC, *Spodoptera exigua*, detoxification enzymes, cuticle super micro structure, cuticular penetration

## Abstract

Beet armyworm, *Spodoptera exigua* (Hübner) is one of the most destructive pests that causes significant losses in crops. Unfortunately, *S. exigua* have developed resistance toward the majority of insecticides. Synergists may provide an important choice to deal with the resistance problems. Dodecyl dimethyl benzyl ammonium chloride (DDBAC) is a cationic surfactant, which displayed enhancement effect when combined with chlorpyrifos against *S. exigua*, giving enhancement factors of 1.50 and 1.57 at the concentrations of 90 and 810 mg L^−1^. In order to clarify the possible mechanisms, we investigate the effects of DDBAC on detoxification enzymes. However, DDBAC showed no inhibition on these enzymes activities. Meanwhile, scanning electron microscope images indicated DDBAC did not affect the cuticle super micro structure of *S. exigua*. The alterations in cuticular penetration rate have also been observed; indeed, it has been suggested that synergism is obtained by an acceleration of insecticide penetration through the cuticle. The chlorpyrifos penetration increased sharply when combined with 90 and 810 mg L^−1^ DDBAC, with only 12.6 and 8.5% of the initial chlorpyrifos recovered by external rinsing after 8 h. In contrast, when there was no DDBAC, more than 23.3% of the initial dose was recovered after 8 h.

## Introduction

Beet armyworm, *Spodoptera exigua* (Hübner) (Lepidoptera: Noctuidae), is a serious and polyphagous pest feeding on many crops, such as asparagus, aubergine, celery, citrus, courgette, lettuce, pepper, tomato, and watermelon. Although *S. exigua* is originally from south-eastern Asia, it is now a worldwide pest that is abundant in North and Central America, Southern Asia, Europe, Africa, and Australia (Smagghe et al., 2003; Lasa et al., 2007; Sántis et al., 2012). *S. exigua* is a pest of complete metamorphosis with a life cycle containing four stages; eggs, five larval instars, pupae, and adults. Larvae feed on both foliage and fruit of crops, and *S. exigua* eats more cabbages than the diamondback moth, *Plutella xylostella* (Linnaeus) (Li et al., 2013). Broad-spectrum insecticides have been widely used singly or in cocktails at weekly intervals to control *S. exigua* infestations (Lasa et al., 2007; Sántis et al., 2012). As a result of the rampant application of insecticides, some *S. exigua* have developed resistance toward different classes of insecticides (Moulton et al., 2000, 2002; Osorio et al., 2008). Resistances of *S. exigua* to a great deal of insecticides have been reported in the United States, in Pakistan, and other countries (Brewer and Trumble, 1989, 1994; Ahmad and Arif, 2010; Ishtiaq and Saleem, 2011; Ishtiaq et al., 2012). Che et al. reported that *S. exigua* were resistant to chlorpyrifos with resistant ratios of 8 to 3,080-fold in China (Che et al., 2013). Even some populations of *S. exigua* are resistant to the newer insecticides, such as chlorantraniliprole, emamectin benzoate, indoxacarb, spinosad, and tebufenozide (Che et al., 2013; Li et al., 2013).

Insecticide synergists make a great contribution to deal with the resistance problems in insecticide applications. For example, piperonyl butoxide (PBO) has been widely used as a synergist for insecticide formulation. It plays an important role in the control of insect pests (Jewess, 2000; Bao et al., 2016). Besides, triphenyl phosphate (TPP) and diethyl maleate (DEM) are also important synergists through inhibiting the detoxification enzymes activities (Wang et al., 2013, 2014). In addition, adjuvants are often used in combination with many classes of pesticides to increase the control efficacy and reduce the application amount (Cocco and Hoy, 2008). Depending on the charge of the hydrophilic group, adjuvants are generally categorized into cationic, anionic, and non-ionic compounds (Olkowska et al., 2011). The cationic adjuvant contains positively charged surface-active portion, for example, salt of a long-chain amine and quaternary ammo-nium chloride. Dodecyl dimethyl benzyl ammonium chloride (DDBAC) is a cationic adjuvant applied to sterilize and remove algae, and is also used in circulating water cooling systems (Wang et al., 2015). Liu et al. reported that cationic DDBAC could enhance the insecticidal activity of beta-cypermethrin, chlorpyrifos, and chlorfenapyr against *S. exigua* and *Helicoverpa armigera*, but the specific synergistic mechanism remains unclear (Liu et al., 2011).

In order to clarify the possible mechanisms by which DDBAC increased the toxicity of chlorpyrifos against *S. exigua*, we investigate the effects of DDBAC on detoxification enzymes and target enzyme viz., cytochrome P450 monooxygenases, glutathione S-transferases, carboxylesterase, and acetylcholinesterase. We also conducted cuticle super micro structure assays for *S.exigua* treated with DDBAC. In addition, the cuticle penetration of chlorpyrifos applied with DDBAC was also studied.

## Insects

*S. exigua* larvae were obtained from scallion fields in Taian, Shandong Province, China, in 2013. *S. exigua* larvae were reared in the laboratory on an artificial diet (corn meal, soybean flour, and yeast as major ingredients) (27 ± 1°C, 14:10 h light: dark photoperiod). Adults were reared under the same conditions at 50–75% RH and fed with 10% honey solution. Males and females were placed in 20 × 40 cm cages for mating and egg-laying.

### Chemicals

Ninety-seven percentage chlorpyrifos were obtained from DowAgro Sciences, 45% DDBAC (Dodecyl dimethyl benzyl ammonium chloride) was purchased from Shandong (Taihe) chemical factory, China. 1,2-Dichlora-4-nitrobenzene (DCNB), p-nitrophenol (PNP), α-naphthyl acetate (α-NA), 1-Naphthol, fast blue B salt and coomassie brilliant blue G-250 were purchased from Shanghai chemical factory, China. Bovine serumalbumin (BSA) were purchased from Wuhan technology Co. Ltd, China. Glutathione (GSH) was purchased from Wuhan alpha biotech Co. Ltd. China, and SDS was purchased from Tianjin Kaitong chemical reagent Co. Ltd. China.

### Enhancement effect of DDBAC on chlorpyrifos against *S. exigua*

The toxicity of chlorpyrifos with DDBAC against *S. exigua* third-instar larvae was tested by insect-dip method. The stock solution of chlorpyrifos (50,000 mg L^−1^ in DMSO) was diluted using 0.05% (w/v) triton X-100 aqueous solution containing 0, 10, 90, and 810 mg L^−1^ DDBAC to the desired concentrations. Individual *S. exigua* larvae were dipped in the solutions for 3 s and dried on tissue paper. Afterwards individual *S. exigua* larvaes were transferred to separate clean glass scintillation vials (22-mm diameter) and maintained on an artificial diet at 27 ± 1°C and 14:10 h light: dark photoperiod. All of the bioassays used 5 different insecticide concentrations with three replications per concentration, and each replication included 24 *S. exigua* larvae. Controls were tested with 0.05% triton X-100 aqueous solution using the same methods. Mortalities were assessed after exposure for 48 h, with any larvae not moving when touched with a fine brush considered dead, and the dose response (LC_50_) was calculated.

### Preparation of *S. exigue* homogenate

The *S. exigua* third-instar larvae were treated by insect-dip method with varying concentrations of DDBAC (10, 90, 810 mg L^−1^). And *S. exigua* larvae were collected after exposure for 5 h. *S. exigua* were homogenized on ice with 2 mL of 0.04 M phosphate buffer pH 7.0 (CarE assay), 66 mM phosphate buffer pH 7.0 (GST assay), 0.1 M phosphate buffer pH 7.4 (AchE assay), or 0.1 M phosphate buffer pH 7.6 (containing 1mM EDTA, 0.1mM DTT, 1mM PTU, and 1mM PMSF) (cytochrome P450 assay). After homogenization, the homogenate was centrifuged at 10, 000 rpm for 15 min (4°C). The supernatant was used as enzyme source for the measurement. The amount of protein of the enzyme source was determined by the Bradford method using bovine serum albumin as a standard.

### Carboxyl esterase (CarE) assay

The activity of CarE was analyzed using α-naphthyl acetate as substrate following the method of Van Asperen (Van Asperen, 1962) with slight modification. One milliliter of enzyme was incubated with 5 mL of substrate solution containing 3 × 10^−4^ M α-NA and 1 × 10^−4^ M physostigmine (an inhibitor of acetylcholinesterase), for 30 min at 30°C. One milliliter of distilled water containing 3 mg of fast blue B salt and 36 mg of SDS was added to terminate the reaction. Absorbance at 600 nm was read after 30 min using a Synergy HT multi-mode microplate reader (BioTek, Winooski, VT). The results were expressed as mOD_600_ min^−1^ mg protein^−1^. At least three replicates of enzyme sources were tested and five individuals for each replicate.

### Glutathione S-transferase (GST) assay

The activity of GST was analyzed using DCNB as substrate (Habig et al., 1974). Enzyme solution (0.2 mL) was incubated with 30 mM DCNB (0.1 mL) and 66 mM phosphate-buffered saline (PBS 2.4 mL, pH 7.0). Enzyme activity was measured using a Synergy HT multi-mode microplate reader at 340 nm (27°C) using the kinetic mode for 5 min. The results were expressed as mOD_340_ min^−1^ mg protein^−1^. Three replicates of enzyme sources were tested and five individuals for each replicate.

### P450 monooxygenase assays

The activity of cytochrome-P450-dependent monooxygenase (P450) was analyzed using p-NA as the substrate in accordance with the method of Hansen et al. (Hansen and Hodgson, 1971), with slight modification. 200 μL mixture containing 100 μL of p-NA (2 mM), 10 μL of NADPH (9.6 mM), and 90 μL of the enzyme stock solution, was incubated at 27°C in air (2 min). Enzyme activity was measured using a Synergy HT multi-mode microplate reader at 405 nm (27°C) using the kinetic mode for 30 min. The results were expressed using mOD_405_ min^−1^ mg protein^−1^. Three replicates of enzyme sources were tested and five individuals for each replicate.

### Acetylcholinesterase (AchE) assay

The activity of AchE was measured using acetylthiocholine iodide as substrate following the method of Ellman (1961). 0.05 mL of 75 mM acetylthiocholine iodide and 0.1 mL of 0.1 M DTNB were prepared, then 0.05 mL of enzyme was added and incubated with shaking at 27°C (15 min). This reaction was terminated by adding 0.1 mL of 1 × 10^−3^ physostigmine. Absorbance at 410 nm was read using a Synergy HT multi-mode microplate reader. The results were expressed as mOD_410_ min^−1^ mg protein^−1^. Three replicates of enzyme sources were tested and five individuals for each replicate.

### Cuticle penetration of chlorpyrifos

The experiments were carried out to determine whether DDBAC could increase the cuticle penetration rate of chlorpyrifos. The dorsal surface of third-instar larvae was dosed individually with 0.5 μL of chlorpyrifos (408.5 mg L^−1^, corresponding to the LC_50_ value) containing 0, 10, 90, and 810 mg L^−1^ DDBAC using a micro-applicator (Burkhard Manufacturing Ltd, UK), and ~500 larvae were included in each concentration. The treated *S. exigua* were collected at 0, 1, 2, 4, and 8 h after treatment. And 20 survivors were pooled to provide one sample for each time point. Treatments were replicated three times. To collect surface chlorpyrifos, *S. exigua* samples were washed in acetone (3 × 1 mL), after which, the eluate was dried under pure N_2_ at 20°C and then resuspended in 1 mL of 100% methanol. Each sample was determined using ultra-high-performance liquid chromatography coupled to tandem mass spectrometry (UHPLC-MS/MS) (Waters Corp., USA) with an electrospray ionization source in positive mode (ESI+). Chlorpyrifos was assayed using the method of Tian et al. (2016). Dynamic of relative percentage of chlorpyrifos penetrated into the 3th instar larvae body of *S. exigua* was calculated by A = (1−B/C) × 100%. (A-The cuticle penetration rate, B-Amount of chlorpyrifos measured on the surface of 20 larvae, C-Amount of chlorpyrifos dripped on the surface of 20 larvae).

### Scanning electron microscopy (SEM)

Individual *S. exigua* third-instar larvae were dipped in the DDBAC solutions (10, 90, and 810 mg L^−1^) for 3 s. Control were tested with water solution using the same methods. The treated larvae were transferred to the glass tubes (9 cm length, 2 cm diameter) and provided artificial diet. Three larvae per concentration were collected after 24 h, immediately fixed in 4% glutaraldehyde in 0.05 M phosphate buffer (pH 7.3) kept 30 min, and then processed as follows. They were rinsed in a phosphate buffer three times for 15 min. Finally, the cuticle were dehydrated in a graded alcohol series of 30, 50, 70, 80, 90% in each case for 15 min, and in 100% twice for 30 min. Ethanol was then replaced by liquid carbon dioxide and samples dried using HCP-2 critical point drying apparatus (Hitachi, Japan). Immediately before the observation, a 10 nm coating of gold-palladium was made using a sputter coater. The cuticle wax layer was examined in a Hitachi S-4800 cold field emission scanning electron microscope (Hitachi, Japan).

### Data analysis

The LC_50_ values were calculated by software SPSS 13.0 (SPSS Inc., Chicago, IL, USA). Data are presented as mean ± standard error (SE). Data were statistically analyzed using one-way analysis of variance (ANOVA) followed by Fisher's LSD test and *t*-test (*P* < 0.05) (Wang et al., 2009) Enhancement factors were calculated as the LC_50_ of chlorpyrifos alone/LC_50_ in presence of DDBAC.

## Results

### The toxicity of DDBAC and its effects on chlorpyrifos

The toxicity of chlorpyrifos plus DDBAC against *S. exigua* was shown in Table 1. Chlorpyrifos showed low toxic to the strain of *S. exigua* raised in the laboratory, and the LC_50_ value was as high as 408.5 mg L^−1^. DDBAC had much lower toxicity against *S. exigua*. It is not toxic to *S. exigua* at the concentrations of 90 and 810 mg L^−1^, but it could increase the toxicity of chlorpyrifos at these two concentrations. The LC_50_ values of chlorpyrifos in presence of DDBAC (at the concentrations of 90 and 810 mg L^−1^) were 272.9 and 260.6 mg L^−1^, giving enhancement factors of 1.50 and 1.57, respectively. These results indicated that DDBAC at the concentrations of 90 and 810 mg L^−1^ can significantly synergized the effect of chlorpyrifos. But no synergistic effect was found when DDBAC at 10 mg L^−1^. And DDBAC at the concentrations of 90 and 810 mg L^−1^ could cause the reduction of applied chlorpyrifos by 33.3 and 36.3% respectively.

**Table 1:** Enhancement effects of DDBAC to chlorpyrifos on the third-instar larvae of *S.exigua*.

	**Concentration of DDBAC (mg L^−1^)**	**Slope ± SE**	**LC_50_(mg L^−1^)**	**95% Fiducial limits**	**χ^2^(*df*)**	***P* values**	**EF^a^**
DDBAC			>3,000				
Chlorpyrifos		1.90 ± 0.19	408.52	339.83 ~ 487.59	3.42(13)	0.99	
Chlorpyrifos + DDBAC	10	1.85 ± 0.19	370.82	305.99 ~ 443.94	3.51(13)	0.99	1.10
	90	1.92 ± 0.19	272.90	229.01 ~ 327.44	7.07(13)	0.90	1.50
	810	1.88 ± 0.19	260.55	218.02 ~ 313.00	4.74(13)	0.98	1.57

a *Enhancement Factor (EF) = LC_50_ for chlorpyrifos alone/LC_50_ in presence of DDBAC*.

### Effects of DDBAC on the detoxification enzymes and target enzyme activities of *S. exigua*

**Figure 1:**
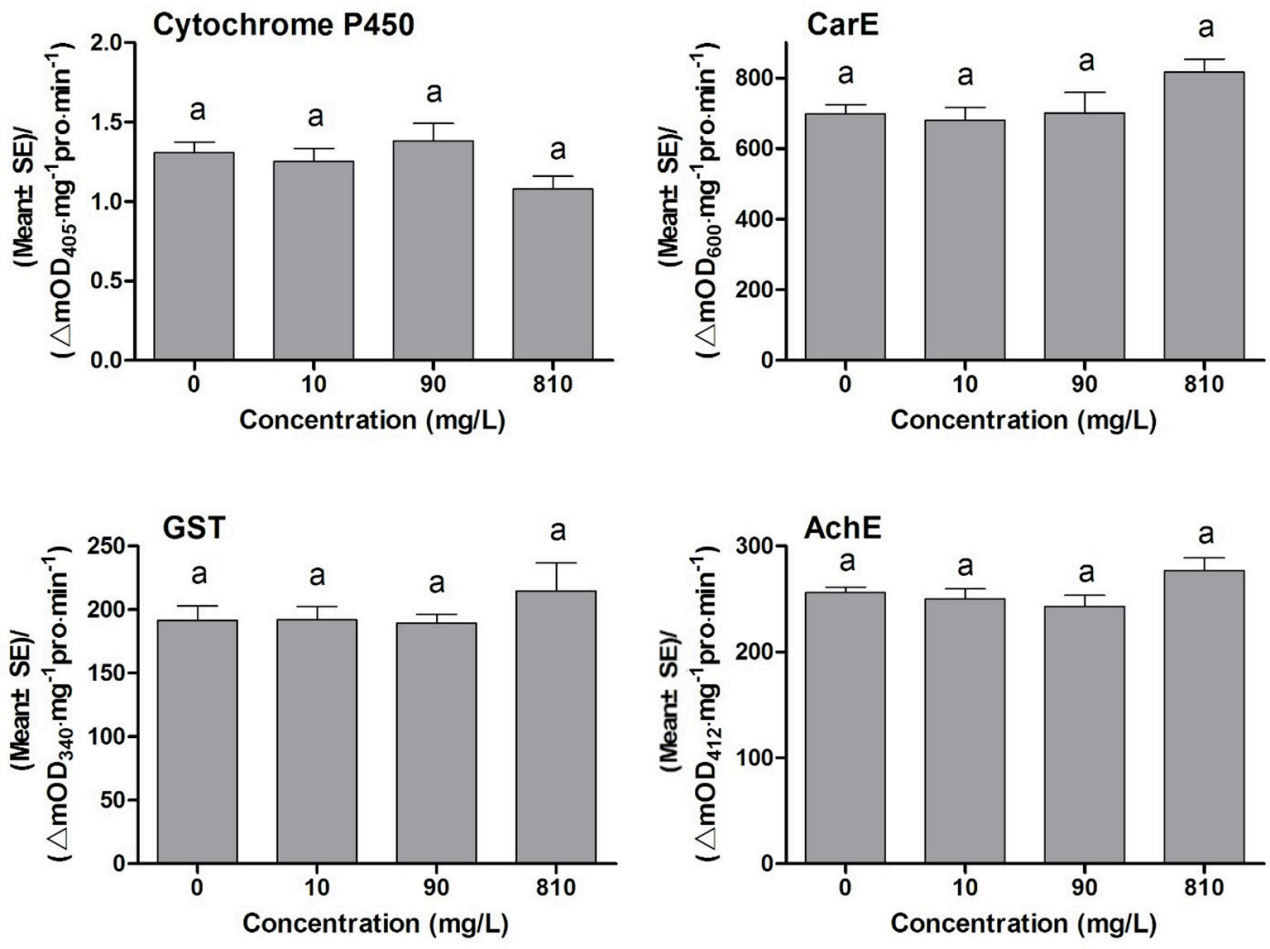
The activity of cytochrome P450, CarE, GST, and AchE of *S. exigua* treated with DDBAC (0, 10, 90, and 810 mg/L). The results were presented as means ± SE. Same letters above the bars indicated no significant differences at *P* < 0.05 (one-way ANOVA, Duncan's multiple range test).

Many synergists exhibit synergistic effects on the toxicity of insecticides, and the synergistic mechanism of most synergists is through inhibiting the detoxification eznymes activities, such as PBO inhibiting P450 monooxygenases activities, TPP inhibiting ESTs activities, and DEM inhibiting GSTs activities (Bao et al., 2016). To find out whether DDBAC showed inhibition effects on the activities of detoxification enzymes, DDBAC was applied to insects and then the changes in enzyme activities were determined. The activities of detoxification enzymes and target enzyme viz., cytochrome P450, CarE, GST, and AchE in *S. exigua* treated with DDBAC were presented in Figure 1. No significant difference (*F* = 2.19, *df*_1_ = 3, *df*_2_ = 8, *P* = 0.17) was observed in cytochrome P450 activity among three DDBAC treatments and control. Although the activities of CarE, GST and AchE were increased by DDBAC at the dose of 810 mg L^−1^, no statistically significant changes in CarE (*F* = 2.30, *df*_1_ = 3, *df*_2_ = 8, *P* = 0.16), GST (*F* = 0.73, *df*_1_ = 3, *df*_2_ = 8, *P* = 0.56), and AchE (*F* = 2.36, *df*_1_ = 3, *df*_2_ = 8, *P* = 0.15) were detected. The results clearly revealed that DDBAC showed no inhibition on these enzymes activities and it had a distinct mode of action as a synergist.

### Penetration of chlorpyrifos combined with DDBAC

**Figure 2:**
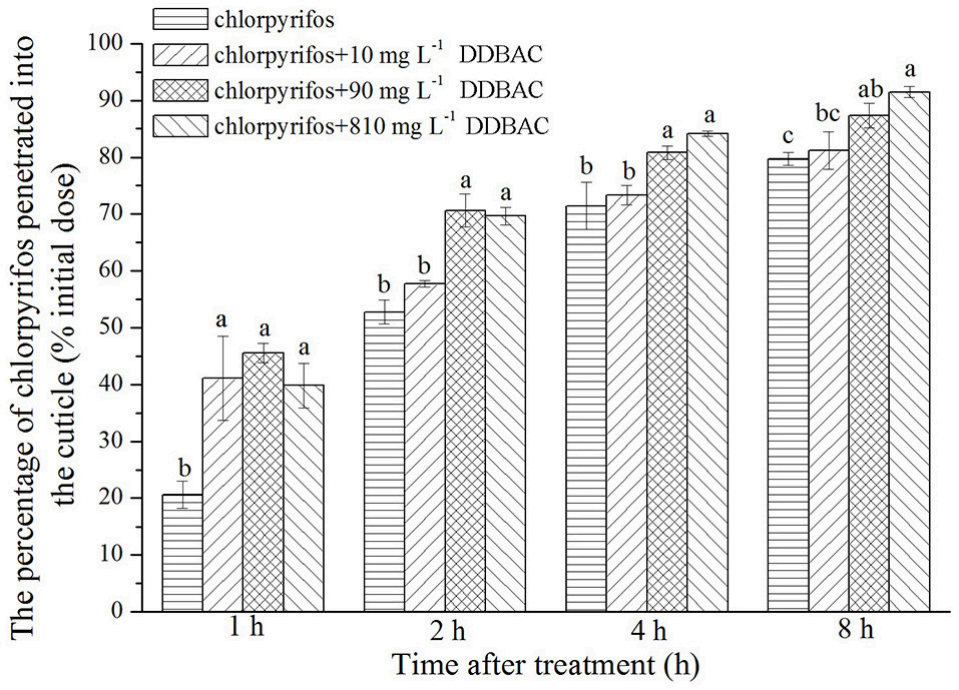
Result of *in vivo* penetration of chlorpyrifos with DDBAC. Graph showed the percentage of the initial chlorpyrifos dose penetrated into the cuticle at different time points after application. The results were presented as means ± SE. Different letters above the bars indicated significant differences at *P* < 0.05 (one-way ANOVA, Duncan's multiple range test).

An *in vivo* penetration assay revealed DDBAC significantly increased the penetration of chlorpyrifos through the cuticle of *S. exigua* (see Figure 2). At the initial time point (1 h), the rate of chlorpyrifos penetration across the cuticle was greatly enhanced by DDBAC, with no notable differences among three concentrations of DDBAC. However, after 2 h, only 90 and 810 mg L^−1^ DDBAC could increase the chlorpyrifos penetration, and the penetration amount increased sharply (to 70.6 and 69.6%), a trend that continued throughout the time course of the experiment, with only 12.6 and 8.5% of the initial chlorpyrifos dose recovered by external rinsing after 8 h. In contrast, when there was no DDBAC, the levels of chlorpyrifos penetrated across the cuticle increased at a much slower rate, with only 20.6% of the applied dose penetrated after 1 h and more than 23.3% of the initial dose recovered after 8 h.

### Effects of DDBAC on the cuticle super micro structure of *S. exigua*

**Figure 3:**
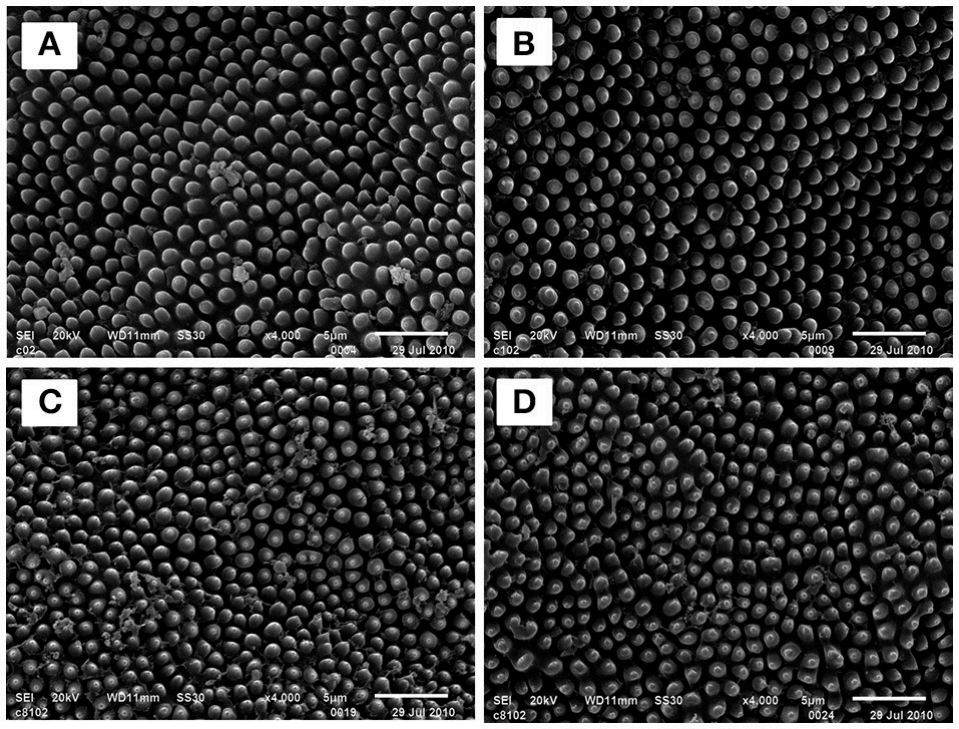
Scanning electron micrographs of the cuticle wax layer of *S. exigua* treated with DDBAC 4000 × **(A)** section was the control *S.exigua* 4000 × **(B–D)** sections represented *S. exigua* treated with DDBAC at dosage of 10, 90, and 810 mg L^−1^, respectively.

Scanning electron microscope (SEM) observation of *S. exigua* treated with DDBAC revealed its effects on the morphology of the cuticle wax layer (Figure 3). SEM images showed that there was no difference in *S. exigua* cuticle super micro structure among the treated groups (DDBAC at dosage of 10, 90, and 810 mg L^−1^, respectively) and control. This result indicated that DDBAC did not affect the cuticle super micro structure of *S. exigua*.

## Discussion

There is a contradiction between the necessity of pesticides application and its potential adverse effects. Reducing pesticide application, increasing unit activity and selectivity, and reducing pesticide resistance development rate are important ways to deal with this dilemma. And the application of synergists is an effective strategy to increase pesticides toxicity and reduce pesticides usage amount (Bao et al., 2016). Here, we found that DDBAC, a cationic adjuvant, showed significant enhancement effect on chlorpyrifos, although DDBAC itself was inactive against insect pests at these synergistic concentrations.

The impact of adjuvants on pesticides has been widely investigated in the past. Adjuvants might impact the physicochemical properties, such as viscosity and surface tension with relevance for spray atomization and influence the contact angle, droplet retention and deposit structure on the target (Stock and Briggs, 2000; Wang and Liu, 2007; Melo et al., 2015). Furthermore, some adjuvants could improve the control efficacy of pesticides through increasing their bio-availability on the target surfaces, for example, through raised or faster penetration (Kudsk et al., 1991; Zabkiewicz et al., 1993). The improvement of cuticular penetration may be directly through improved deposit formation or structural and chemical changes on target cuticle (Bukovac and Petracek, 1993; Kraemer et al., 2009). Improving the efficacy of insecticides through adjuvants is much more complex than increasing the activity of herbicides (Knowles, 2001; Melo et al., 2015). So only few studies are available for insecticides, although many studies have exhibited fairly well the impact of adjuvants on herbicides and fungicides. Our study indicated that DDBAC can enhance the insecticide activity and possibly reduce the dose for insecticide application.

PBO is the most universal insecticide synergist, which has played an important role in the control of malaria mosquito vectors and insect vectors of other diseases. And PBO is also commonly used in formulations for the control of urban insect pests (Jewess, 2000; Bao et al., 2016). The synergism for PBO stems from its abilities to inhibit two major metabolic enzymes, P450s and non-specific esterases, and to enhance the cuticular penetration of insecticides into insects (Bingham et al., 2011). However, DDBAC did not exhibit inhibition on the activity of three important detoxification enzymes, P450 monooxygenases, glutathione S-transferases, and carboxylesterase. The results indicated that DDBAC might work in a different way from inhibiting the activities of detoxification enzymes.

SEM images indicated that DDBAC did not affect the cuticle super micro structure of *S. exigua*. However, DDBAC significantly improved the penetration of chlorpyrifos through the cuticle of *S. exigua*. DDBAC have a high infiltration capacity and its positive charge can combine with the negative charge in insect cuticle. It may also act as solvent to dissolve insecticides and as surfactant on the “waxy” cuticle to enhance the speed of insecticide arriving at the target site. The quicker the penetration of insecticide, the lesser the opportunity for detoxification (Sun and Johnson, 1972). So, the toxicity of insecticide can be enhanced by DDBAC.

Moreover, application of DDBAC is an important way to lower the resistant levels of insecticides, because insecticide resistance can be attributable to reduced cuticular penetration. Insecticide resistance through decreased cuticular penetration has been reported in several insect pests including housefly [*Musca domestica* (L.), red flour beetle (*Tribolium castaneum*), cotton bollworm (*Helicoverpa armigera*), German Cockroach (*Blattella germanica*), corn earworm [*Helicoverpa zea* (Boddie)], diamondback moth [*P. xylostella* (L.)], *Heliothis virescens* and *Myzus persicae* (Ahmad et al., 2006; Puinean et al., 2010). Szeicz et al. reported insecticides penetrated more rapidly and reached higher concentrations in internal tissues of larvae of the susceptible tobacco budworm (Szeicz et al., 1973). And the penetration of deltamethrin into resistant *H. armigera* was obviously slower than into susceptible individuals over 24 h. It took only 1 h for 50% penetration of the applied deltamethrin in the susceptible strain but 6 h for the resistant strains (Ahmad et al., 2006).

In conclusion, DDBAC accelerated the penetration of chlorpyrifos through the cuticle to enhance insecticide toxicity against *S. exigua*. So DDBAC can be used in insect pests control to increase the toxicities of conventional insecticide and reduce the application amount of pesticides ingredient.

## Author contributions

Conceived and designed the experiments: LC, HY, DY, CR, and WM. Performed the experiments: LC. Analyzed the data: LC, DY. Wrote the paper: LC, HY, DY, CR, and WM.

### Conflict of interest statement

The authors declare that the research was conducted in the absence of any commercial or financial relationships that could be construed as a potential conflict of interest.

## References

[B1] Ahmad M. Arif M. I. (2010). Resistance of beet armyworm *Spodoptera exigua* (Lepidoptera: Noctuidae) to endosulfan, organophosphorus and pyrethroid insecticides in Pakistan. Crop Prot. 29, 1428–1433. 10.1016/j.cropro.2010.07.025

[B2] Ahmad M. Denholm I. Bromilow R. H. (2006). Delayed cuticular penetration and enhanced metabolism of deltamethrin in pyrethroid-resistant strains of *Helicoverpa armigera* from China and Pakistan. Pest Manage. Sci. 62, 805–810. 10.1002/ps.1225 16649192

[B3] Bao H. B. Shao X. S. Zhang Y. X. Deng Y. Y. Xu X. Y. Liu Z. W.. (2016). Specific synergist for neonicotinoid insecticides: IPPA08, a *cis*-neonicotinoid compound with a unique oxabridged substructure. J. Agric. Food Chem. 64, 5148–5155. 10.1021/acs.jafc.6b01512 27281691

[B4] Bingham G. Strode C. Tran L. Khoa P. T. Jamet H. P. (2011). Can piperonyl butoxide enhance the efficacy of pyrethroids against pyrethroid-resistant *Aedes aegypti*? Trop. Med. Int. Health 16, 492–500. 10.1111/j.1365-3156.2010.02717.x 21324051

[B5] Brewer M. J. Trumble J. T. (1989). Field monitoring for insecticide resistance in beet armyworm (Lepidoptera: Noctuidae). J. Econ. Entomol. 82, 1520–1526. 10.1093/jee/82.6.1520

[B6] Brewer M. J. Trumble J. T. (1994). Beet armyworm resistance to fenvalerate and methomyl: resistance variation and insecticide synergism. J. Agric. Entomol. 11, 291–300.

[B7] Bukovac M. J. Petracek P. D. (1993). Characterizing pesticide and surfactant penetration with isolated plant cuticles. Pest. Sci. 37, 179–194. 10.1002/ps.2780370212

[B8] Che W. N. Shi T. Wu Y. D. Yang Y. H. (2013). Insecticide resistance status of field populations of *Spodoptera exigua* (Lepidoptera: Noctuidae) from China. J. Econ. Entomol. 4, 1855–1862. 10.1603/EC13128 24020303

[B9] Cocco A. Hoy M. A. (2008). Toxicity of organosilicone adjuvants and selected pesticides to the Asian citrus psyllid (Hemiptera: Psyllidae) and its parasitoid *Tamarixia radiata* (Hymenoptera: Eulophidae). Florida Entomol. 91, 610–620. 10.1653/0015-4040-91.4.610

[B10] Ellman G. L. (1961). A new and rapid colorimetric determination of acetycholine-esterase activity. Biochem. Pharmacol. 7, 88–95. 10.1016/0006-2952(61)90145-9 13726518

[B11] Habig W. H. Pabst M. J. Jakoby W. B. (1974). Glutathione S-transferases, the first enzymatic step in mercapturic acid formation. J. Biol. Chem. 249, 7130–7139. 4436300

[B12] Hansen L. G. Hodgson E. (1971). Biochemical characteristics of insect microsomes: N- and O-demethylation. Biochem. Pharmacol. 20, 1569–1572. 10.1016/0006-2952(71)90285-1 4399525

[B13] Ishtiaq M. Saleem M. A. (2011). Generating susceptible strain and resistance status of field populations of *Spodoptera exigua* (Lepidoptera: Noctuidae) against some conventional and new chemistry insecticides in Pakistan. J. Econ. Entomol. 104, 1343–1348. 10.1603/EC10383 21882702

[B14] Ishtiaq M. Saleem M. A. Razaq M. (2012). Monitoring of resistance in *Spodoptera exigua* (Lepidoptera: Noctuidae) from four districts of the Southern Punjab, Pakistan to four conventional and six new chemistry insecticides. Crop Prot. 33, 13–20. 10.1016/j.cropro.2011.11.014

[B15] Jewess P. J. (2000). Piperonyl butoxide: the insecticide synergist. Integr. Pest Manage. Rev. 5, 147–148. 10.1023/A:1009697512567

[B16] Knowles A. (2001). Adjuvants for agrochemicals. Pest. Outlook 12, 183–184. 10.1039/b108611c

[B17] Kraemer T. Hunsche M. Noga G. (2009). Cuticular calcium penetration is directly related to the area covered by calcium within droplet spread area. Sci. Hortic. 120, 201–206. 10.1016/j.scienta.2008.10.015

[B18] Kudsk P. Mathiassen S. K. Kirknel E. (1991). Influence of formulations and adjuvants on the rainfastness of maneb and mancozeb on pea and potato. Pest. Sci. 33, 57–71. 10.1002/ps.2780330107

[B19] Lasa R. Pagola I. Ibañez I. Belda J. E. Williams T. Caballero P. (2007). Efficacy of *Spodoptera exigua* multiple nucleopolyhedrovirus as a biological insecticide for beet armyworm control in greenhouses of southern Spain. Biocontrol Sci. Technol. 17, 221–232. 10.1080/09583150701211335

[B20] Li H. Jiang W. Zhang Z. Xing Y. R. Li F. (2013). Transcriptome analysis and screening for potential target genes for RNAi-mediated pest control of the beet armyworm, *Spodoptera exigua*. PLoS ONE 8:e65931. 10.1371/journal.pone.0065931 23823756 PMC3688801

[B21] Liu Y. Q. Zhang G. S. Zhou C. Wang W. Hu Y. P. Mu W. (2011). Synergistic action of cationic adjuvants 1227 and C _8−10_ and the silicone adjuvant Breakthru S240 to three insecticides. Acta Entomol. Sin. 54, 902–909. 10.16380/j.kcxb.2011.08.003

[B22] Melo A. A. Usano-Alemany J. Guedes J. V. C. Hunsche M. (2015). Impact of tank-mix adjuvants on deposit formation, cuticular penetration and rain-induced removal of chlorantraniliprole. Crop Prot. 78, 253–262. 10.1016/j.cropro.2015.09.021

[B23] Moulton J. K. Pepper A. D. Dennehy T. J. (2000). Beet armyworm (*Spodoptera exigua*) resistance to spinosad. Pest Manage. Sci. 56, 842–848. 10.1002/1526-4998(200010)56:10<842::AID-PS212>3.0.CO;2-H

[B24] Moulton J. K. Pepper D. A. Jansson R. K. Dennehy T. J. (2002). Pro-active management of beet armyworm (Lepidoptera: Noctuidae) resistance to the tebufenozide and methoxyfenozide: baseline monitoring, risk assessment, and isolation of resistance. J. Econ. Entomol. 95, 414–424. 10.1603/0022-0493-95.2.414 12020022

[B25] Olkowska E. Polkowska Z. Namiesnik J. (2011). Analytics of surfactants in the environment: problems and challenges. Chem. Rev. 111, 5667–5700. 10.1021/cr100107g 21744834

[B26] Osorio A. Martínez A. M. Schneider M. I. Díaz O. Corrales J. L. Avilés M. C.. (2008). Monitoring of beet armyworm resistance to spinosad and methoxyfenozide in Mexico. Pest Manage. Sci. 64, 1001–1007. 10.1002/ps.1594 18418831

[B27] Puinean A. M. Foster S. P. Oliphant L. Denholm I. Field L. M. Millar N. S.. (2010). Amplification of a cytochrome P450 gene is associated with resistance to neonicotinoid iInsecticides in the aphid *Myzus persicae*. PLoS Genet. 6:e1000999. 10.1371/journal.pgen.1000999 20585623 PMC2891718

[B28] Sántis E. L. Hernández L. A. Martínez A. M. Campos J. Figueroa J. I. Lobit P.. (2012). Long-term foliar persistence and efficacy of spinosad against beet armyworm under greenhouse conditions. Pest Manage. Sci. 68, 914–921. 10.1002/ps.3250 22262560

[B29] Smagghe G. Pineda S. Carton B. Estal P. D. Budia F. Viñuela E. (2003). Toxicity and kinetics of methoxyfenozide in greenhouse-selected *Spodoptera exigua* (Lepidoptera: Noctuidae). Pest Manage. Sci. 59, 1203–1209. 10.1002/ps.756 14620046

[B30] Stock D. Briggs G. (2000). Physicochemical properties of adjuvants: values and applications. Weed Technol. 14, 798–806. 10.1614/0890-037X(2000)014[0798:PPOAVA]2.0.CO;2

[B31] Sun Y. P. Johnson E. R. (1972). Quasi-synergism and penetration of insecticides. J. Econ. Entomol. 65, 349–353. 10.1093/jee/65.2.349 5016658

[B32] Szeicz F. M. Plapp F. W. Vinson S. B. (1973). Tobacco budworm: penetration of several insecticides into the larva. J. Econ. Entomol. 66, 9–15. 10.1093/jee/66.1.9 4690304

[B33] Tian F. J. Liu X. G. Xu J. Dong F. S. Zheng Y. Q. Hu M. F. (2016). Simultaneous determination of phoxim, chlorpyrifos, and pyridaben residues in edible mushrooms by high-performance liquid chromatography coupled to tandem mass spectrometry. Food Anal. Methods 9, 2917–2924. 10.1007/s12161-016-0490-x

[B34] Van Asperen K. (1962). A study of housefly esterases by means of a sensitive colorimetric method. J. Insect Physiol. 8, 401–416. 10.1016/0022-1910(62)90074-4

[B35] Wang C. J. Liu Z. Q. (2007). Foliar uptake of pesticides - present status and future challenge. Pest. Biochem. Phys. 87, 1–8. 10.1016/j.pestbp.2006.04.004

[B36] Wang D. Qiu X. H. Ren X. X. Zhang W. C. Wang K. Y. (2009). Effects of spinosad on *Helicoverpa armigera* (Lepidoptera: Noctuidae) from China: tolerance status, synergism and enzymatic responses. Pest Manage. Sci. 65, 1040–1046. 10.1002/ps.1794 19533589

[B37] Wang S. P. Hu X. X. Meng Q. W. Muhammad S. A. Chen R. R. Li F.. (2013). The involvement of several enzymes in methanol detoxification in Drosophila melanogaster adults. Comp. Biochem. Physiol. B Biochem. Mol. Biol. 166, 7–14. 10.1016/j.cbpb.2013.05.008 23751173

[B38] Wang Y. N. Zhang Y. Li X. Sun M. Z. Wei Z. Wang Y.. (2015). Exploring the effects of different types of surfactants on zebrafish embryos and larvae. Sci. Rep. 5:10107. 10.1038/srep10107 26053337 PMC4459078

[B39] Wang Z. L. Zhao Z. Abou-Zaid M. M. Arnason J. T. Liu R. Walshe-Roussel B.. (2014). Inhibition of insect glutathione s-transferase (gst) by conifer extracts. Arch. Insect Biochem. Physiol. 87, 234–249. 10.1002/arch.21192 25270601

[B40] Zabkiewicz J. A. Stevens P. J. G. Forster W. A. Steele K. (1993). Foliar uptake of organosilicone surfactant oligomers into bean leaf in the presence and absence of glyphosate. Pest. Sci. 38, 135–143. 10.1002/ps.2780380208

